# Life cycle assessment and multicriteria decision making analysis of additive manufacturing processes towards optimal performance and sustainability

**DOI:** 10.1038/s41598-025-92025-5

**Published:** 2025-07-11

**Authors:** Jayant M. Raut, Prashant B. Pande, Kamlesh V. Madurwar, Rajesh M. Bhagat, Satyajit S. Uparkar, Nilesh Shelke, Haytham F. Isleem, Arpita, Vikrant S. Vairagade

**Affiliations:** 1 Department of Civil Engineering, Yeshwantrao Chavan College of Engineering, Nagpur, Maharashtra 441110 India; 2 Department of Civil Engineering, Priyadarshini Bhagwati College of Engineering, Nagpur, Maharashtra 440024 India; 3 Department of Computer Science and Applications, Ramdeobaba University, Nagpur, Maharashtra 440013 India; 4 Symbiosis Institute of Technology, Nagpur Campus, Symbiosis International (Deemed) University, Pune, Maharashtra 440008 India; 5 Department of Computer Science, University of York, York, YO10 5DD UK; 6 Department of Biosciences, Saveetha School of Engineering, Saveetha Institute of Medical and Technical Sciences, Chennai, 602105 India; 7 Department of Civil Engineering, Priyadarshini College of Engineering, Nagpur, Maharashtra 440019 India

**Keywords:** Sustainable construction, Life cycle assessment, Additive manufacturing, Gaussian process regression, Multi-criteria decision analysis, Particle swarm optimization, Engineering, Materials science, Mathematics and computing

## Abstract

The pressing need for sustainable construction materials and processes has been driving research into the optimum environmental and economic efficiency of Additive Manufacturing (AM). Most models available for Life Cycle Assessment (LCA), however, do not capture the dynamism of real-time data and the existing levels of uncertainty, and decision-making frameworks are not adaptive to evolving sets of criteria. In this paper, these described limitations are addressed through the introduction of an integrated approach that couples predictive Life Cycle Assessment (LCA) with Gaussian Process Regression (GPR), dynamic decision criteria weighting via Stochastic Forest for Multi-Criteria Decision Analysis (MCDA), and multi-objective optimization using Particle Swarm Optimization (PSO). In this study, GPR-based predictive LCA is conducted using historical and real-time environmental data for modeling impact categories of CO_2_ and energy use. This methodology makes estimates of not only the mean impact but also allows quantification of the uncertainties through confidence intervals and dynamic LCA. Stochastic Forest algorithm will enhance the traditional MCDA by weighting decision criteria like cost, environmental impact, and durability, in a more dynamic manner aligning to real-time manufacturing performance for better decision-making. Further, PSO will optimize material and process parameters to balance the multiple objectives of material strength, energy efficiency, and cost-effectiveness. In this way, this integrative novel approach of machine learning with bioinspired optimization contributes to the sustainability of AM. Experimental results prove that predictive accuracy can be achieved up to 85–90% by GPR, which reduces material wastage by 12%. By using Stochastic Forest, an improvement in decision accuracy can be attained to the extent of 15–20%, together with a cut in costs of about 10%. For its part, PSO optimizes design and manufacturing parameters for the materials, raising their efficiency by 10–15%, while the energy consumption goes down by 8–12%. The next framework is an important step toward integrating the steps reviewed for the development of sustainable Additive Manufacturing practices. This framework overcomes the present limitations of the LCA model by introducing dynamic predictive modeling using Gaussian Process Regression, real-time adaptive decision-making through Stochastic Forest, and multi-objective optimization through Particle Swarm Optimization. This integration of the techniques in the framework would help address the real-time data and uncertainties that are inherent for adaptive and sustainable solutions in additive manufacturing processes.

## Introduction

The conventional construction and materials technology-based global construction industry finally comes under increasing pressure for implementing sustainable practices in the wake of the immense environmental impacts. An area of increased focus in Additive Manufacturing (AM), in comparison to conventional construction techniques, concerns the possibility of exploiting it in a way that will increase material efficiency, save energy, and reduce waste. Benefits include the ability to create complex geometries with precise material placement; hence, leading to much optimization with respect to resource usage. While AM holds the potential for significant strides in the gamut of benefits, making it sustainable in the true sense focuses on the life cycle from material extraction to manufacture to the product’s end-of-life. Although major developments in technologies for additive manufacturing have been carried out, the current approaches to LCA and LC optimization are indeed very limited. Traditional LCAs are usually static, providing a snapshot of environmental impacts at a point in time and without considering real-time changes regarding manufacturing processes or changes in environmental conditions. These lack the capability to predict future environmental impact on the basis of dynamic input data and can rarely allow the guiding of such decisions sustainably over the long term. Furthermore, many frameworks readily accessible today, which apply in material and process selection in AM, are very inflexible in the sense that they rely on fixed criteria weights and do not change over real-time performance data samples but over historical data samples. This diminishes the scheme to make poor choices where it does not balance between sustainability and being representative and cost-effective in performance. Furthermore, the optimization algorithms often attended to in AM design and manufacture work singularly, specifically on the objective of minimal cost or focus on only the maximum material strength to the exclusion of other factors, not considering the multi-objective real-world problems where trade-offs are always made between environmental, economic, and performance factors.

This paper presents a new iterative framework for sustainable AM, which integrates advanced machine learning and optimization techniques. In this paper, we demonstrate that Gaussian Process Regression, Stochastic Forest for multicriteria decision analysis, and Particle Swarm Optimization are used to establish a dynamic adaptive model for an adaptive model that updates itself over the real-time estimation data samples. With an explicit consideration of reducing environmental impacts, improving material efficiency, and cost minimization, while quantifying the uncertainties of predictive modeling, the proposed approach aims at the optimization of material design and manufacturing processes simultaneously. Use of the Gaussian Process Regression enables this model with the capability to model uncertainties and generate smooth predictive insight into the life cycle impacts of AM materials and processes. Contrary to ordinary regression methods, for the GPR setting, the relationship between input variables, for instance, consumption of materials, energy, and emissions, and results of the LCA, is assumed to be a stochastic process. Therefore, it is possible to carry out mean predictions and corresponding confidence intervals, allowing for a much better insight into possible environmental impacts over sets of temporal instances. Real-time environmental parameters, such as temperature and humidity, are integrated into GPR during dynamic LCA in varying conditions. The dynamic approach in this respect is important to achieve the sustainability of AM in the long term, as it is capable of making ongoing modifications to the various AM processes for real-time data with the aim of reducing material wastage and saving energy.

A Stochastic Forest is complemented to GPR to increase the intelligent system’s capability by online recomputing and adapting weights of decision criteria based on the real-time sample performance data. It is perfectly right for AM because many criteria have to be considered at the same time: cost and environmental benefit with durability. Traditional MCDA approaches often rely on expert-derived weights that remain fixed throughout the decision-making process. However, such fixed weights are not very realistic for reflecting perhaps the changed importance of some criteria once new data have influenced the results. This paper presents a more flexible data-driven approach to MCDA using the advanced ability of the Stochastic Forest algorithm to deal with big data, discovering the most important factors in the multicriteria decision process, updating with the evolution of the manufacturing process. This is because it can provide decision-makers with a dynamic weighting in such a way that moving the sliders would automatically normalize the performance indicators under the consideration of the decision-maker. This dynamic weighting ensures decision-makers could thereby continuously optimize during the making of their choices, balancing trade-offs between sustainability, performance, and cost-effectiveness as conditions change. Finally, the multi-objective optimization problem in AM is tackled utilizing Particle Swarm Optimization (PSO). PSO is a bio-inspiration algorithm for simulating the social behavior of birds flocking or fish schooling to explore a solution space. This is the optimization of material composition and manufacturing parameters simultaneously with the view of a number of objectives minimizing unfavorable environmental impacts and maximizing material strength. Unlike traditional optimization algorithms, which deal with a single objective, the use of PSO explores the tradeoffs between conflicting goals using a solution that would be fair, sustainable, and more balanced. PSO is also very adaptive: every particle holds a dynamically changing position based on its previous experience and the experience it gathers from neighboring particles. This allows the optimization process to be consistent and enables its results to improve continuously with the introduction of new data over sets of chronological instances. Therefore, a novel integrated framework in this paper comprises important progress in this direction, as it tackles the current endeavors in trying to practice sustainable AM. With GPR, Stochastic Forest for MCDA, and PSO embedded in a closed loop iterative-based process, continuous optimization of material design and manufacturing parameters can be rendered possible based on real samples of material design and manufacturing data along the AM process. The rhythmic property of this framework guarantees that decision-making will be conducted in light of alterations in environmental conditions and manufacturing performance to yield more sustainable outcomes over the life cycle of the AM products. First experimental results prove the concept to be valid, while at the same time improvement in material efficiency, energy reduction, and improvement in decision-making accuracy enables the entire AM process to be more sustainable and economic. This should motivate the construction sector to put into practice these types of advanced applications, together with the kind of methodology and techniques in machine learning and optimization, as those that were outlined. The proposed iterative framework, next to covering the gaps of the existing LCA and decision-making models, is poised to lead the practices of additive manufacturing into better flexibility and sustainability. This work of research is beneficial for both academicians and industry professionals in creating the most effective, eco-friendly, and cost-efficient materials and processes to construct any product.

The framework will combine GPR, Stochastic Forest, and PSO in a synergistic way to address the complexity of sustainable additive manufacturing. GPR will be the predictive backbone, making use of historical and real-time data to model environmental impacts like CO_2_ emissions and energy consumption with quantified uncertainty. In such a case, it may be the environmental impact weight that increases when the scenarios are of higher emissions, and thus the decisions maintain sustainability focus. It ensures to dynamically reflect the changes in the manufacturing environment because of balancing the cost-performance with sustainability in decisions through Stochastic Forest. This integration is finally completed by PSO, where it makes an optimization for the material configurations and process parameters, based on the outputs from GPR and decision criteria weights from Stochastic Forest. For example, when GPR gives a prediction for sharp growth of energy usage, PSO then changes the thickness of a layer or the content of recycled material so as not to disturb its action. So there is feedback—PSO makes further optimizations on predictive models in GPR; fine-tuned predictions by GPR are used to create feedback for additional optimization. Stochastic Forest allows such changes to be dynamically weighted according to priorities in real-time; it is thus that the framework can iterate and converge on optimal solutions. This connected process ensures that the model will be both robust and adaptable to real-world uncertainties and can provide practical, sustainable solutions in the AM Process.

A combined and adaptive framework of Predictive Life Cycle Assessment (LCA), Gaussian Process Regression (GPR), Stochastic Forest, and Particle Swarm Optimization (PSO) might be capable of addressing the complexity of sustainable additive manufacturing (AM). Predictive LCA powered by GPR is used as the foundational base of modeling environmental impacts like CO_2_ emissions and energy consumption. GPR combines historical and real-time data to generate dynamic predictions with uncertainty quantification through confidence intervals. In GPR, the predictions produce forward-looking evaluations of the environmental effects throughout the AM process, giving much-needed information on which of these aspects influence subsequent decision-making and optimization. For example, if GPR projects an increase in emissions based on specific material configurations, this information is immediately available for further evaluation by the decision-making algorithm process. The GPR-driven LCA is complemented by Stochastic Forest and PSO, dynamic refinement of the decision-making and optimization processes. The weights on the decision criteria of cost, durability, and environmental impact are tuned based on real-time data such that the decision-making process responds to changing priorities in the Stochastic Forest. These weights feed into PSO, which runs multi-objective optimization to trade off competing goals of reduced emissions and energy use against maximizing material performance. Here, dynamic synergy is a system where every component enhances the effectiveness of others. This framework integrates the predictive capability of GPR, the adaptive decision-making ability of Stochastic Forest, and the optimization strength of PSO to provide robust, sustainable, and practical solutions for AM processes.

### Motivation and contribution

Under this regard, the primary motivation for this research is that the current scenario about the construction industry points at a growing need for sustainable practices within the sector, which remains one of the largest contributors to environmental degradation through excessive consumption of resources, high energy use, and generation of huge amounts of wastes. This will open up an opportunity where such challenges could be dealt with because there will be potential for additive manufacturing that is proving to be a conceivable alternative to the traditional ways of construction. However, despite the potential of AM, methodologies in place for assessing and optimizing its environmental impacts are not adequately equipped to address dynamic and real-time data and the associated uncertainties that characterize a manufacturing process. Traditional methods of LCA are rather static in nature, by and large, and bereft of adaptability, and decision-making frameworks mostly have no regard for any time. Varying nature of criteria weights as a function of performance data samples. This research will, therefore, try to fill these gaps through the development of a comprehensive iterative method that integrates GPR for predictive LCA, stochastic forest for dynamic MCDA, and PSO for multi-objective optimization.

The contributions of this paper are threefold. First, this research advances the state-of-the-art field of LCA with the integration of GPR, a machine learning technique that allows modeling uncertainties and makes dynamic predictions of the environmental impacts with confidence intervals. As such, it allows for real-time updates of the LCA so that decision makers can continuously monitor and adjust processes in light of environmental changes or market dynamics. Second, integration of stochastic forest within the MCDA framework introduces a new way of dynamically updating decision criteria weights based on samples of real-time performance data. This guarantees that the decision-making process is based on data and adaptable to dynamic changes, making the accuracy of the decision solutions high, relevant to the materials and manufacturing processes selected, in the additive manufacturing process. Out of these, multi-objective optimization employing PSO substantiates substantial improvement in attaining the optimization of processes and materials for AM: it can balance objectives such as maximum performance and minimum environmental impacts to gain solutions that are more sustainable and cost-effective. These contributions collectively provide a robust framework for sustainability optimization for AM in construction, with humongous potential to reduce resource consumption, energy use, and waste, improved accuracy of decisions, and material efficiency.

### Replicability of the process

Specific parameters for both the PSO and Stochastic Forest algorithms for improving the study’s replicability are discussed further. In implementation of the PSO algorithm, the swarm size was set at 100 particles to ensure reliable exploration of solution space. A linear decrease function was applied throughout iterations, thereby initializing the inertial weight with a value at 0.7 and its subsequent linear variation to achieve effective convergence towards the optimum. The acceleration coefficients for cognitive and social were chosen to be 1.5 and 2.0, respectively, balancing personal best positions against global best positions that guide particle movements. Updating the velocity for each particle includes stochastic components which are drawn uniformly from the interval [0,1] and ensure enough randomness to avoid being trapped in a local optimum. The algorithm was run for 500 iterations based on the termination criterion of stabilizing the values of the objective functions in process. The decision criteria in the Stochastic Forest algorithm were dynamically weighted using metrics based on the reduction of impurities such as the Gini index. The algorithm uses 200 decision trees with the construction of every tree through the use of bootstrapped samples of training data samples. For each decision at a split of the nodes, some randomness in the decision criteria was undertaken. This is controlled through setting the max features parameter to √n, where nnn refers to the number of features. The weights for the criteria were iteratively changed with real-time performance data from the additive manufacturing processes. The normalization of the sum to be equal to one ensured perfectly balanced trade-offs across the optimization process and was computed as follows: Specification of these parameters ensures that the study allows replication of the methodologies by the researchers. PSO and Stochastic Forest were tuned through pilot experiments to achieve a balance between exploration and convergence, and the parameters align with best practices in optimization and machine learning for additive manufacturing process.

## Review of existing models for additive manufacturing

Discussions of sustainable construction are part of the fast development in the area of additive manufacturing in order to reduce materials and process-related environmental impacts. The Table 1 shows that it is a multidisciplinary review of works done. The focus on LCA and optimization algorithms supported by BIMs is a continuation of efforts towards the inclusion of sustainability metrics in construction and manufacturing sectors. Several studies report that the estimation of impact on the environment requires LCA as the fundamental tool. Karunaratne, Dharmarathna, and De Silva^1^ have discussed in detail the principles underlying the South Asian LCA development and provided useful insights into the global warming potential with different construction methodologies. Similarly, Fnais, Rezgui, Petri, et al.^2^, discussed at length about the implementation issues pertaining to LCA in buildings, and their study summarized major barriers involved in the process, such as data availability and methodological limitations. These studies underline an important role of the LCA in sustainable decision making, which at the same time has to point to major deficiencies in the integration of data associated with real-time environmental conditions. Filling such knowledge gaps with robust LCA methodologies, Shen^3^ furthered the study, applying Apriori algorithms in combination with BDAs to identify carbon reduction opportunities in public buildings. Although such approach demonstrated huge potential applied with big datasets in making decisions on sustainability, it is limited to data-rich environments, therefore raising a question about its applicability to contexts where infrastructural data development remains low. Fidan, Aydogan, and Uzal (2024) only used the LCA framework for the extension to the textile industry and elaborated more on social subcategories. Although this work allowed setting new dimensions of sustainability through social impact assessment, it is fairly industry-specific and limits possible cross-sector applications.

**Table 1:** Empirical review of existing methods.

Reference	Method used	Findings	Results	Limitations
Karunaratne, S., Dharmarathna, D. & De Silva, N.^1^	Life cycle assessment (LCA)	Developed a whole building LCA tool for South Asia	Quantified global warming potential effectively	Limited to South Asian climatic and regulatory contexts
Fnais, A., Rezgui, Y., Petri, I. et al.^2^	LCA	Explored challenges and future research directions for LCA in buildings	Identified key barriers in the application of LCA	Lacked specific case studies and practical solutions
Shen, X.^3^	Apriori algorithm, big data analysis	Applied Apriori algorithm for carbon reduction in public buildings	Demonstrated significant reductions in carbon emissions	Focused mainly on data-rich environments, limiting generalizability
Fidan, F.Ş., Aydoğan, E.K. & Uzal, N.^9^	LCA	Conducted a comprehensive social impact analysis of the textile industry	Provided detailed insights into social subcategories of LCA	Industry-specific, limiting cross-sector applicability
Basile, V., Petacca, N. & Vona, R.^10^	Literature review	Reviewed circularity measures in Life Cycle Management	Summarized key circularity indicators and metrics	Focused more on theory than practical applications
Mohamed, B., Marzouk, M.^7^	Bibliometric analysis	Analyzed heritage building preservation through bibliometric methods	Highlighted global research trends in heritage preservation	Lacked real-world case studies for validation
Barbero, I., Rezgui, Y., Beach, T. et al.^8^	Social life cycle assessment (SLCA)	Assessed current work in SLCA in the construction sector	Identified future research needs for social sustainability in construction	Social factors are still underexplored compared to environmental ones
Jędrzejczyk, A., Firek, K., Rusek, J. et al.^11^	Convolutional neural network (CNN), SVM	Developed models for predicting damage intensity in masonry buildings	Achieved high accuracy in damage prediction	Focused only on masonry buildings, limiting generalizability
Kong, W., Luo, H., Yu, Z. et al.^12^	Bibliometric analysis	Reviewed global trends in retrofitting buildings for sustainability	Highlighted key areas for economic and environmental improvements	Lacked detailed case studies for specific retrofitting scenarios
Apellániz, D., Alkewitz, T. & Gengnagel, C.^13^	3D business intelligence dashboards	Visualized LCA results using 3D BI dashboards	Improved clarity in interpreting LCA results for decision-makers	Limited interactivity and depth of data exploration
Song, J., Wang, W., Ni, P. et al.^6^	Low-carbon retrofit framework	Proposed a low-carbon retrofit framework for rural buildings	Demonstrated effectiveness in reducing carbon in arid regions	Regional specificity may limit its application elsewhere
Latupeirissa, J.E., Arrang, H.^4^	Building information modeling (BIM)	Evaluated sustainability factors of BIM in project management	Provided insights into the benefits of BIM for lifecycle management	Focused on the Indonesian construction sector
Wang, Z., Hu, L., Huang, X. et al.^14^	LCA	Assessed carbon emissions in power transmission projects	Offered a detailed analysis of the full lifecycle carbon footprint	Focused solely on smart energy systems
Solanki, S.K., Paul, V. & Singh, V.^15^	Maintenance management framework	Developed a maintenance management blueprint for institutional buildings	Improved maintenance efficiency and lifecycle cost reduction	Lacked applicability to non-institutional buildings
Minde, P., Kulkarni, M., Shelake, A.G. et al.^16^	Rework reduction approach	Proposed a systematic approach for reducing rework in precast buildings	Reduced rework instances significantly in case studies	Focused only on the precast industry in India
Olanrewaju, A., Wong, W.F. & Lim, P.I.^17^	Association rule technique	Identified factors affecting maintenance demand in hospital buildings	Uncovered key relationships between building age and maintenance demand	Focused solely on hospital infrastructure
Zhang, Z., Qiu, Y.^5^	Cloud-BIM	Developed a framework for analyzing construction safety of steel structures	Improved safety analysis during the construction phase	Limited application to urban steel structure buildings

This is the sole reason for which optimization algorithms are recently evolving as powerful tools to enhance sustainability in construction and manufacturing processes. Latupeirissa and Arrang^4^ also mentioned that BIM was applied for the construction project management in Indonesia with a significant effort placed to -improve the quality of projects through minimized delays, more efficient use of resources, and enabling better communication among the stakeholders. Zhang and Qiu^5^ further extended the applications into safety in urban steel construction, providing evidence of the effectiveness cloud BIM systems have towards improving the level of safety analysis and the initiated measures during the construction process. However, BIM implementation is still low because of the non-existence of standardized means, practices, or legislation for the regions and sectors, which recommends the necessity for even higher levels of harmonization in the future use of it.

There has been a specific focus of a couple of studies on some sectors, which are seen as worthwhile case examples in contributing to the understanding of sustainable construction practices. For instance, Song et al., Wang, and Ni et al.^6^ formulated a rural residential building low-carbon renovation framework for the arid areas of China, enabling considerable cuts in carbon emissions through targeted interventions. Also, Mohamed and Marzouk^7^ carried out a bibliometric analysis on heritage building preservation that delineated the global trends concerning the use of LCA and other sustainability metrics in heritage projects. Obviously, detailed sector-specific studies like the above not only provide critical insight into contextual challenges and opportunities in improving guard sustainability, but their applicability is not always well transferred to other industrial and regional frameworks. From these findings, the trend that clearly emerges is the integration of social sustainability and environmental sustainability indicators within construction and manufacturing processes. These observations are consistent with those of Barbero et al.^8^, who state that “the growing interest in SLCA in the construction sector, virtually mandates the need for much more integrated frameworks covering both environmental and social impacts”. They reflect the continual development of LCA methodologies, which keep on changing and incorporating broader dimensions of sustainability. Nevertheless, the complexity of the integration of the social issues is still a challenge in the LCA framework, as is the case with limited cross-sectorial studies in the textile sector scenarios, such as the case of Fidan, Aydoğan, and Uzal^9^. This research presents evidence concerning radically different methodologies: advanced techniques in machine learning and optimization algorithms, on the one side, and BIM-based systems, on the other, working toward the same greater research aim of enhanced sustainability in construction and manufacturing. The results show that the optimization in materials and processes has evolved very significantly, but there are still some key challenges that exist for the most part: real-time data integration, harmonization of sustainability metrics across industries, and social dimensions of sustainability.

Table 1 clearly emphasizes the message of sustainability, optimization and, digital technologies within the construction and manufacturing sectors. Main findings indicate that the sustainable front is being pushed by the advanced methodologies, such as life cycle assessment. Means at the moment that these innovative tools are marching into every sector for receiving greater efficiency in resource use, a reduced negative impact on the environment, and an improved structure of performance. Basically, one of the strongest recurring themes across the reviewed papers is the development and need to develop better-integrated sustainability assessment tools. Over time, LCA has proved to be a pertinent tool in estimating construction projects’ environmental impacts, among others. However, data availability, regional specification, and the methodological constraining have taken out some of its potential. For instance, Karunaratne, Dharmarathna, and De Silva^1^ make important contributions to LCA in a South Asian setting, though their work tends largely to signal the necessity for frameworks of this kind to be much more easily reconfigurable for use in different climates and under somewhat different regulatory conditions. For instance, commenting on the application of LCA in the building sector on a larger scale, Fnais et al.^2^ stated that future research should overcome barriers to integrating information. The optimization model has been efficient in dealing with the multi-objectiveness of sustainability-related constructional challenges. Even though these methods greatly assist in gaining an understanding of the associated trade-offs in terms of sustainability and performance, practical application is bounded by the concerning integration complexity of large dataset scale and real-time environmental conditions.

One of the brilliant improvements is in BIM as a computing instrument to improve sustainability in the construction life cycle. It just shows that studies like that by Latupeirissa and Arrang^4^, Zhang and Qiu^5^, assert that through BIM, resource applications become more effective, communication becomes better, and safety analysis becomes better. However, despite the prominence of BIM in recent construction, this application is still not centralized, mainly related to non-standardization problems of interoperability within regions and sectors. Future studies on BIM must be oriented to the harmonization of its use and increasing the likelihood of potential benefit realization across different contexts. Research in construction is definitively shifting toward social sustainability. For example, Barbero et al.^8^ have said that sustainability does not pertain solely to the natural setting but also to social life. The increased attention to the social life cycle assessment in construction projects is a call to arms, if you will, and indicative of sophisticated recognition that sustainability encompasses a social element as much as it does an ecologic dimension. The other challenge is to incorporate the social factors into the traditional LCA frameworks, and much work has to be done in the development of sound methodologies that can effectively capture the entire spectrum of sustainability impacts for various scenarios. This review represents the progress of developing sustainability for construction and manufacturing with the aid of LCA together with optimization algorithms and digital tools, such as BIM. They can be combined to provide a very powerful framework for the treatment of complex, multi Vective challenges in themes of sustainable development; however, a number of key challenges have shown themselves, namely in data integration, real-time adaptability, or including social sustainability metrics. Future research, however, should particularly deal with the efforts to overcome these drawbacks in the development of more or less holistic and integrated approaches regarding sustainability evaluation and optimization, as the field itself evolves.

## Optimization model for sustainable additive manufacturing

**Fig. 1:**
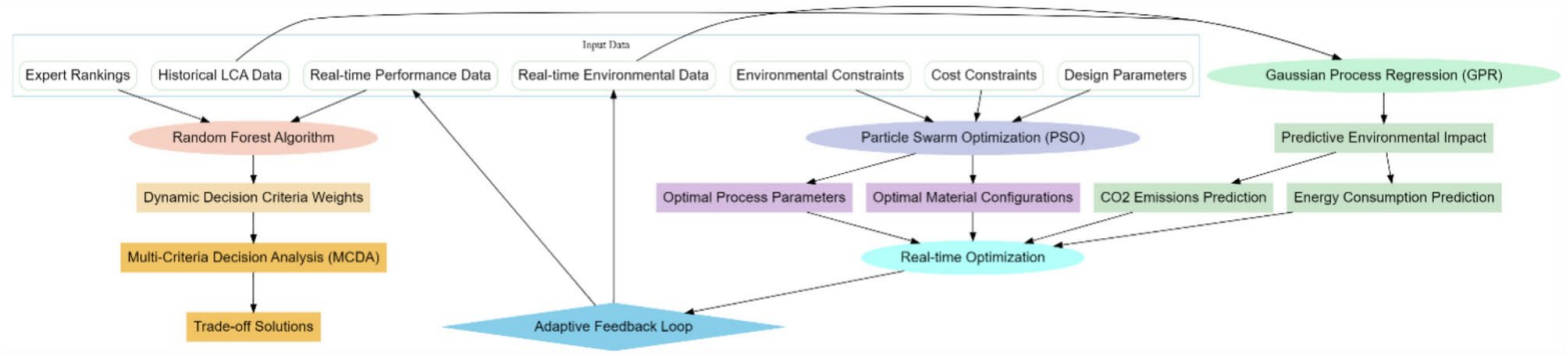
Model architecture of the proposed analysis process.

This section focuses on the designing of an iterative method based on Gaussian process regression and particle swarm optimization to be applied for analysis in additive manufacturing as a means of surmounting low efficiency and high complexity issues with the existing methods for sustainable additive manufacturing operations. Figure 1: A Predictive Life Cycle Analysis is implemented using the Gaussian Process Regression methodology, which initiates with a probabilistic framework that integrates historical life cycle data and real-time environmental and operational parameters. GPR is used because it has the intrinsic ability to model complex, nonlinear relationships while accounting explicitly for uncertainties. This would be critical in additive manufacturing life cycle analysis, where variability in process parameters and material use, or environmental conditions, may exert large effects on outcome variables like energy consumption and CO_2_ emissions across temporal instance sets. In GPR, the relationship between input variables and output variables is modeled with a Gaussian distribution. Let X = {× 1, × 2,…,xn} be the set of input variables, and let y = {y1,y2,…,yn} represent the corresponding outputs. The intuitive concept behind GPR is to place a prior over all possible functions which may explain the relation between the sets X and y. This prior is a Gaussian Process, defined by a mean function, m(x), and a covariance function, k(x, x′), via Eq. 1,

1 $$f\left(x\right)\sim GP\left(m\left(x\right),k\left(x,{x}{\prime}\right)\right)$$

The covariance function k(x,x′), also known as the kernel, governs the smoothness of the functions that the GPR can model. The used kernel is the squared exponential kernel, given via Eq. 2,

2 $$k\left( {x,x\prime } \right) = \sigma f^{2} \exp \left( { - \frac{1}{{2*l^{2} }}\left| {x - x\prime } \right|^{2} } \right)$$

where, $\sigma {f}^{2}$ represents the variance of the function, and ℓ is the characteristic length scale, controlling how far apart inputs can influence each other. Such a kernel function will let GPR capture smooth variations of environmental impacts over time while allowing for the correlation between different inputs such as temperature, humidity, and resource availability. Prediction under GPR then consists simply of conditioning the Gaussian Process on the observed data to obtain a posterior distribution over functions that are coherent in both the prior and data samples. Given a set of observed inputs, X, and corresponding outputs, y, the posterior distribution at a new input x ∗ will also be Gaussian with predictive mean, μ(x ∗), and variance, σ2(x ∗), given via operations 3 & 4,

3 $$\mu \left(x*\right)=k\left(x*,X\right){\left[K\left(X,X\right)+\sigma {n}^{2}I\right]}^{-1}y$$

4 $${\sigma }^{2}\left(x*\right)=k\left(x*,x*\right)-k\left(x*,X\right){\left[K\left(X,X\right)+\sigma {n}^{2}I \right]}^{-1}k\left(X,x*\right)$$

where, K(X,X) is the covariance matrix of the training inputs, σn2 is the noise variance and I is the identity matrix in the process. These operations are the backbone to GPR and thus, the prediction mechanism of the system relies on it. It gives not only the expected mean impact on the environment through the predictive mean but also the levels of uncertainty associated with it via predictive variance levels. In the LCA context, having predictive variance is important since that allows estimation of confidence intervals associated with uncertainty in predictions. One of the major strengths of GPR in this respect is that it can dynamically update the predictions whenever new data are available. This adaptability ensures the relevance of LCA predictions by LCA based on temporal instance sets for a process in an additive manufacturing case where environmental conditions and process parameters may vary. Said another way, whenever new real-time data is gathered with respect to energy use or leftovers, a GPR model can be updated to capture this new trend for a better and more timely prediction of environmental impacts related to the process. Moreover, integral in GPR will be the ability to model cumulative environmental impacts over the life cycle of a material or process. This is captured by integration of the predicted environmental impact over sets of temporal instances in the process.

Let T represent the time horizon of interest, then the cumulative environmental impact, Icumulative, can be modeled via Eq. 5,

5 $$Icumulative=\int T\mu \left(x\left(t\right)\right)\hspace{0.17em}dt$$

These integral operations can therefore capture the total environmental burden, such as cumulative CO_2_ emissions or energy consumption, over the duration of the AM process. Similarly, integrating the predictive variance is able to quantify the uncertainty in this cumulative impact in order to obtain a cumulative uncertainty bound at the process, which informs about the range of impacts that might potentially go with it. The other critical aspect of the GPR-based LCA is how it deals with nonstationary processes, which may involve environmental and operational parameters that cannot be assumed to be time-invariant across temporal instance sets. In this respect, the use of a nonstationary kernel—such as, for instance, the Matérn kernel—empowers the GPR model to track sudden changes in the input variables, and thus makes the LCA predictions resilient under different conditions. The Matérn kernel is defined via Eq. 6,

6 $$k\nu \left(x,{x}{\prime}\right)=\frac{{2}^{1-\nu }}{\Gamma \left(\nu \right)}{\left(\sqrt{2\nu }\frac{\left(x-{x}{\prime}\right)}{l}\right)}^{\nu }K\nu \left(\frac{\sqrt{2\nu }\left(x-{x}{\prime}\right)}{{\ell}}\right)$$

Specifically, when ν is the parameter controlling the smoothness of the function and $K {u}$ is the modified Bessel function, then it allows for more flexible modeling of environmental impacts that have sudden changes or irregular patterns. Justification for the choice of GPR will be by the flexibility and non-parametric nature of this process, coupled with the ability to quantify the uncertainty, which is very important in applications where predictive accuracy and understanding of uncertainty are paramount. Traditional deterministic models cannot deal with intrinsic variability and uncertainties that exist in complex dynamic systems like AM, while GPR excels in these cases by providing mean predictions along with associated confidence intervals during a process.

**Fig. 2:**
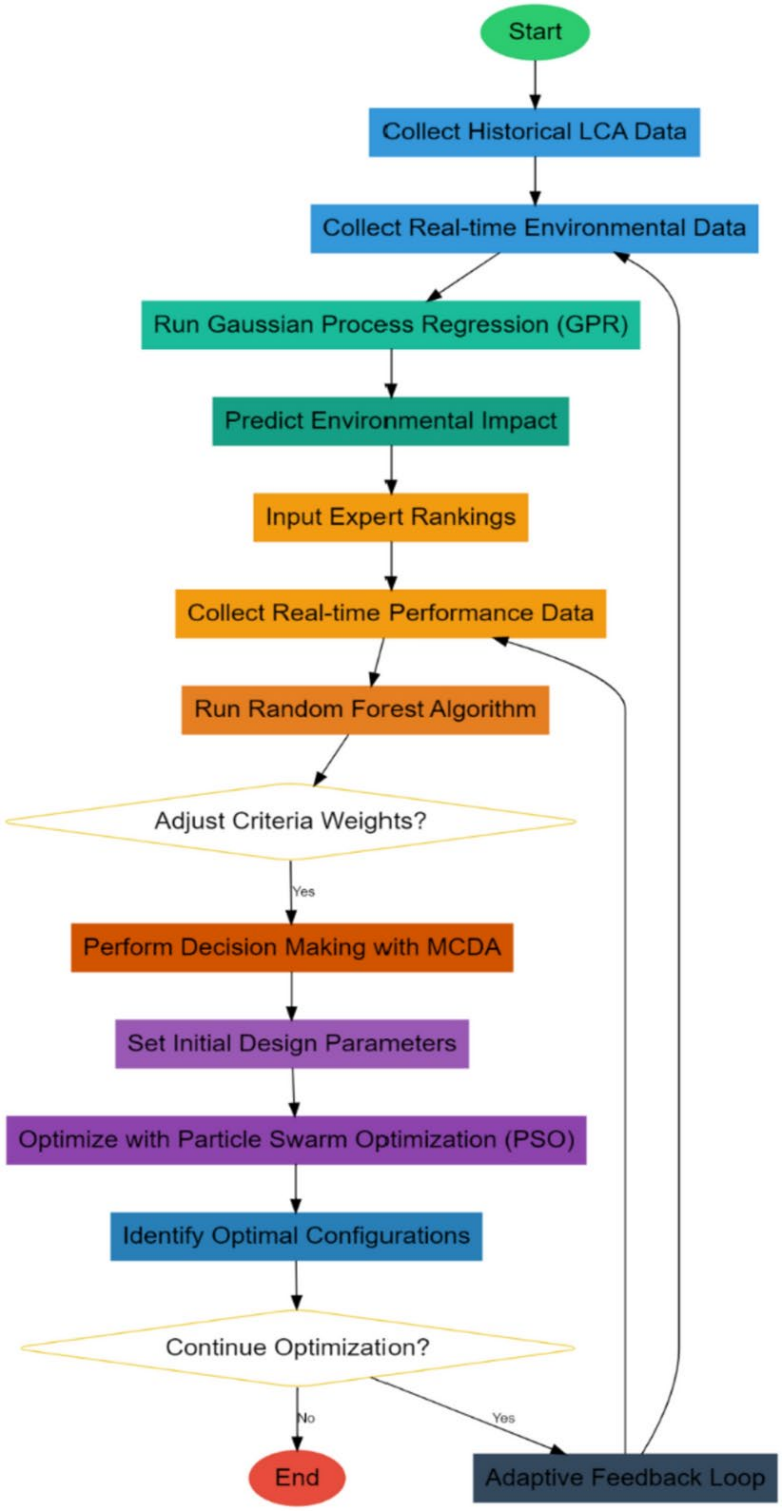
Overall flow of the proposed analysis process.

Figure 2 illustrates the next step toward a decision-making framework using a Stochastic Forest algorithm for Multi-Criteria Decision Analysis in additive manufacturing processes, taking into account the dynamic weighting of sets of decision criteria. The approach deals with some of the intrinsic complexities of AM, where cost, environmental impact, and structural performance are simultaneously in consideration. For instance, one of the powerful ensembles learning methods chosen for this work is the Stochastic Forest, which is robust and resistant to overfitting, thus allowing interpretability in decision-making. This technique will enrich traditional MCDA through its ability to adapt to criteria weights by dynamically changing data-driven insight instance sets with real temporalities, optimizing trade-offs. Stochastic Forests work by creating a large number of decision trees while training, each of them based on a stochastic subset of the data samples. Let $X = \{x_1,x_2,…,x_n\}$ be the input matrix, where each $x_i$ is a vector of the parameters of the decision variables, and let $y = {y_1, y_2,…,y_n}$ be the associated output vector, which denotes the outcome of the decisions. Each of the decision trees, Ti, is trained on a bootstrapped sample of X and y, and at every split, only a stochastic subset of the decision parameters is considered. This will give an ensemble decision tree where the final choice on the decision criterion weights and the solutions to the optimal trade-off are determined by the aggregation of the output from all trees. The basic idea is that each decision tree is constructed by a recursive partitioning of the input space based on decision rules that minimize some measure of impurity; let us use for simplicity the Gini index G levels. For a node t with nt samples, the Gini index is given via Eq. 7,

7 $$G(t)=1-\sum_{i=1}^{C}p{i}^{2}$$

where, pi is the proportion of samples belonging to class i at node t, and C is the number of classes. The entropy is defined via Eq. 8,

8 $$H(t)=-\sum_{i=1}^{C}pi*log\left(pi\right)$$

Thus, at every node, a decision rule that maximizes the decrease in impurity is selected. Therefore, the decision tree will be segmenting data optimally to help differentially infer separate cases of outcomes. Since Stochastic Forest does exactly this by aggregating the output from many trees, the final prediction would be a majority vote in case of classification and average in case of regression, of the outputs from all the individual trees. The main advantage of using Stochastic Forest here is the implicit setting of dynamic importance with respect to a set of decision criteria. This was achieved by considering the decrease in impurity, Gini index, for each criterion in case of splitting at all trees. The importance score of a criterion xj is quantified via Eq. 9,

9 $$Importance\left(xj\right)=\frac{1}{T}\sum_{i=1}^{T}\Delta G\left(i,j\right)$$

where T is the total number of trees in the forest and ΔG(i, j) is the impurity decrease for criterion xj in the Ti sets, and the importance scores are normalized to have a sum of one, which produces a distribution of x that is weighted, representing the influence of a subset of the criteria in decision outcomes. These weights are dynamically set and then utilized to compute the overall utility score for each decision alternative with consideration of the real-time performance data and expert rankings for the decision alternatives. Utility U for an alternative Ak can be denoted by using a weighted sum for its performance across all criteria via Eq. 10,

10 $$U\left(Ak\right)=\sum_{j=1}^{m}wj\cdot fj\left(Ak\right)$$

where wj is the normalized weight of criterion xj, fj(Ak) is the performance score of alternative Ak on criterion xj, and m is the total number of criteria sets. A utility score of this kind can be used for the selection of a tradeoff solution that would optimize the balance between two or more competitive objectives. One of the most vital characteristics of the stochastic forest in this hybrid MCDA framework is its robustness to overfitting. The model smooths out the noise due to individual trees by averaging their outputs in order to make a better prediction. Another salient feature of the ensemble approach ensures that the model remains interpretable—that is, the importance of each criterion can be directly noted right away from the aggregated impurity reductions. Such interpretability is highly valuable in decision-making processes where it is important to understand the rationale behind the chosen weights. Last but not least, the choice of stochastic forest can also be further justified by its ability to complement other components of the AM decision-making framework. While techniques, such as GPR, are in place for the prediction of environmental impacts with quantified uncertainty, Stochastic Forest deals with the optimization of weights of decision criteria in response to such predictions and other samples of performance data. The dynamic weighting mechanism that Stochastic Forest embodies assures continuous updates of the decision-making process when new data become available for adaptive and informed decision-making, by which it reflects the current insight into the process. Moreover, the integrative nature of Stochastic Forest will help to easily include samples for real-time performance data and expert rankings. For example, in case one expert feels a certain impact is highly prioritized to the environment, such input will be combined with real-time data about energy consumed and emissions in order to adjust the weights accordingly. This feature can make the process of decision-making more responsive and adaptable, especially in a rapidly evolving additive manufacturing field.

Finally, PSO integrates particle swarm optimization to optimize material configurations and manufacturing parameters in additive manufacturing through the effective exploration of multi-dimensional solution space balancing multiple objectives. The PSO is a population-based stochastic optimization algorithm inspired by the social behavior in natural systems like birds flocking or fish schooling. It also provides inherent flexibility and nice convergence properties, so it fits perfectly with the complex and multi-objective nature of the optimization problems encountered in AM, having minimization targets for the environmental impacts and the costs, and maximization targets for performance metrics such as material strength and energy efficiency. PSO begins with the initialization of a population (swarm) of candidate solutions called particles. Every particle represents one possible configuration of material properties and process parameters within the process.

These could be, for instance, material composition or layer thickness, energy consumption, and emitted pollutants. Let xi(t) be the position of particle iii in iteration t; this would be a vector of design parameters in multi-dimensions. Given a particle, it has a corresponding, velocity vi(t) which determines the particle’s movement inside the solution spaces. The update operations for the velocity and position of particle i are provided via operations 11 & 12,

11 $$vi\left(t+1\right)=wvi\left(t\right)+c1r1\left(pi\left(t\right)-xi\left(t\right)\right)+c2r2\left(g\left(t\right)-xi\left(t\right)\right)$$

12 $$xi\left(t+1\right)=xi\left(t\right)+vi\left(t+1\right)$$

Here, v _i_ (t + 1) is the new velocity of the particle; w is the inertia weight, which controls how much influence the old velocity has on the new one; c _1_ and c _2_ are acceleration factors weighing the influence of the best-known position of the particle itself, p _i_ (t), and the global best-known position g(t), respectively; and r _1_ and r _2_ represent stochastic numbers drawn from a uniform distribution in the range [0,1] so that some stochasticity will be added to the update process. One could view the velocity equation as a balance of three forces: the previous velocity of the particle, which describes the tendency for the particle to keep moving in its current direction, the attraction to its own best-known position, which enables it to return to a known good solution, and the attraction to the swarm’s best-known position, which enables convergence to the global best solution. The objective function f(xi) evaluates the quality of a particle’s position, considering desired outcomes that are typically minimization of energy usage E(xi), minimization of environmental impacts C(xi)—CO_2_ emissions, and maximization of received levels of material performance P(xi). Using Eq. 13, an optimization task could then be formulated.

13 $$Opt=\stackrel{x}{\text{min}}\alpha 1E(x)+\alpha 2C(x)-\alpha 3P(x)$$

where α1, α2, and α3 are the weight coefficients, which balance the different objectives related to energy use, environmental impact, and material performance. These weights are tuned based on the relative importance of each objective in the given specific context of AM. Some of the strengths of PSO include its ability to handle multiple objectives, either through the aggregation into a single weighted objective function, as above, or by maintaining a set of Pareto frontiers representing trade-offs between conflicting objectives. If using the former of the two approaches, the weighted sum allows decision makers to be biased toward certain outcomes, like environmental sustainability, over others, like cost minimization. The Pareto front approach, on the other hand, avails one with the opportunity to look through a spectrum of optimal solutions, hence offering flexibility in the picking of configurations based on various sets of criteria. Indeed, PSO has already been proved to work out quite well in convergence toward optimal configurations, resulting in reduced material usage and energy consumption yet yielding equivalent or improved material performance. The inertia weight in the velocity update equation is usually reduced over time; that is to say, it is to get smaller in value to force convergence as the swarm gets closer to the global optimum. This adaptivity is one of the major reasons for selecting PSO, since it can aggressively explore the solution space at early stages and subsequently fine-tune solutions in later phases of the optimization process. Moreover, one of the inherent strengths of PSO is its strength in navigating high-dimensional and nonlinear search spaces, making it well-suited to support other machine learning techniques engaged in the AM process, such as GPR. Where GPR offers predictions with uncertainty quantification for life cycle impacts, PSO optimizes the process parameters in keeping with those predictions, enabling a feedback loop in which optimization is now informed continually by real-time samples of environmental and performance data. This synergy assures that the optimization is not biased to go after the optimum solutions in a fixed space, but rather renormalizes continuously in the evolving conditions of the manufacturing process. The dynamic nature of PSO allows for the progressive refinement of solutions over time, especially in new constraints or objectives appear. For instance, as new environmental regulations or cost concerns arise, the algorithm adapts the optimization criteria in a way that sustains the AM process objectives; and the update operations in velocity and position, having within them the ingredients of PSO, are stochastic and therefore bring to the algorithm flexibility and robustness. The algorithm of Particle Swarm Optimization, therefore, constitutes an effective and adaptive way to optimize material configurations as well as process parameters in the field of additive manufacturing. In addition, considering multiple objectives, e.g., minimizing energy consumption, minimizing environmental impacts, and maximizing the performance of the material, PSO is assured to ensure that the process for additive manufacturing is not only effective but also sustainable. The mathematical formulation at the core of PSO, particularly in its velocity and position update operations, lets it grasp the inherent spirit of social behavior and adaptation. This allows the algorithm to efficiently probe the solution spaces while refining them simultaneously. Integrating PSO within the larger decision-making framework of AM presents a potent route toward sustainable manufacturing.

It is such dynamic adjustments to decision criteria in the Stochastic Forest algorithm that play a central role in trading off the issues of cost versus sustainability in additive manufacturing scenarios as developed in the real world. The recalculation of the weights accorded to cost, environmental impact, and performance then sets a balance in the trade-offs between conflicting priorities in the determination carried out by the algorithm. For instance, the weight on the measurement on cost will be escalated by the algorithm if very high material costs have resulted in the strategy for production to still be economical but not at the expense of too much loss in sustainability. Conversely, if the objective is to preserve, say, to comply with regulatory requirements in terms of decreased CO_2_ emissions, then the weight for environmental impact can be more substantial, thus allowing the framework to balance its current focus on emission reduction with appropriate cost boundaries. An algorithm such as Stochastic Forest would adjust its weight on recycled content, yet still take into consideration other sustainability metrics, like energy efficiency. Such concessions might yield virgin material utilization on a temporary basis but would assure lesser general environmental impacts in energy-optimized process parameters. It is the adaptation that the prime need lies in practical applications wherein stringent frameworks for decision making fail to identify nuances of operation dynamics. The dynamic trade-off between cost and sustainability would be the basis through which manufacturers would meet their long-term efficiency and environmental requirements, though they would be in a position to address short-term challenges as well in process.

A more adapted solution would be a combination of predictive LCA, Gaussian Process Regression (GPR), Stochastic Forest, and PSO, which would give broad applicability in AM situations for adaptability purposes toward multiobjective optimization for any real-time input. Therefore, in the field of biomedical-related activities, one could think of optimizing the production of a patient-specific implantation of a particular prosthetic within the frame of such a solution. With the availability of real-time data of the material properties such as biocompatibility and mechanical strength, GPR would be able to predict the impacts on the environment and operation depending upon the variations of different material configurations. Decisions are dynamic with priorities set up by taking into account material costs without a compromise in performance. PSO produces optimal outcomes with parameters including thickness of layer and energy consumed. Such a technique does not only offer efficiency but also ensures that the production process adheres to really tight medical and sustainability standards.

This framework can be applied to optimize high-performance components for the aerospace industry, which emphasizes weight optimization and durability. Predictive LCA may be used in real time under various production conditions to evaluate the environmental footprint of materials like advanced polymers or metal alloys. Emissions of CO_2_ and energy consumption trade-offs can be modeled using GPR. Dynamic adjustment of the decision weights by Stochastic Forest is applied on durability and energy efficiency. The PSO is further optimizing design parameters based on objectives like minimization of weight and maximum strength. Such decisions are refined in an iterative fashion even while operational conditions are in a change process. These examples show the flexibility of the framework to handle industry-specific requirements, making it a powerful tool in advancing sustainable practices across diverse AM applications. In this section, we would elaborate on the efficiency of the proposed model in relation to different metrics and compare it with existing models in various scenarios.

## Comparative result analysis

This research put in place a setup that embeds predictive life cycle analysis, multi-criteria decision analysis, and optimization algorithms in the comprehensive framework of an additive manufacturing process. All the experiments were executed on a real-world dataset, which comprises a historical LCA dataset—material consumption, energy usage, associated emissions, and waste generation data collected from earlier additive manufacturing projects. These historical samples were used as a seed to create future predictions of the environmental impacts by the GPR model, including uncertainty quantification. Streaming of real-time environmental data, such as temperature, humidity, energy consumption rate, resource availability, etc., has been fed into dynamic updating. The following set of multi-criteria decision parameters were used with the Stochastic Forest-based MCDA: cost: $50–$500 per unit; environmental impact in terms of production of CO_2_ in kg per kg of material; durability given by tensile strength ranging from 50 to 200 MPa; ranking by experts, which involves ranking the materials by domain specialists. The expert rankings were collected from five experts in the industry, who have a sound knowledge of sustainable AM. These were mapped against a five-point Likert scale, in which 1 equates to “low importance” and 5 corresponds to “critical importance”. Real-time data on the performance of the manufacturing processes, such as build rate, material efficiency usage, and power usage, was then continuously fed to the model at build rates of between 70 and 90%, with energy use between 10 and 30 kWh per build. The experimental work in this paper makes use of the Ecoinvent database, arguably among the broadest life cycle inventory datasets for sustainability research. Ecoinvent provides very detailed environmental data covering a large variety of materials, energy sources, and processes from industries like manufacturing, construction, and waste management. It has data for inputs, outputs, and associated emissions for every process. This dataset includes metrics on CO_2_ emissions, material consumption, energy use, and resource depletion. Datasets used from Ecoinvent in this research were on environmental impacts of additive manufacturing materials like metals and polymers, and energy consumption data for a number of manufacturing techniques. These data samples enabled the GPR model to simulate the influence of environmental impacts by various material compositions and process parameters on real sets of temporal instances. Due to its fine granularity and global extent, the dataset was very suitable for modeling dynamic LCA and thus accurate region-specific predictions of environmental impacts, further increasing the confidence and relevance of the results obtained in this study.

The PSO algorithm was thus initialized with material composition, layer thickness, and energetic use constraints based on a dataset representative of a multi-material AM setup. In this study, the experimental design is targeted to optimize material configurations that balance sustainability and performance while reducing cost and environmental impact. Sample values assigned to these design parameters varied between 0.05 to 0.5 mm in layer thickness, material compositions with a percentage of virgin and recycled materials—in this case, 20 to 80% recycled content—and the energy usage constraint from 15 to 25 kWh per kilogram of material processed. Other environmental constraints added were maximum CO_2_ that could be emitted, ranging between 100 and 500 kg per build, while the cost constraint was put at a maximum of $100 per kilogram of material. The swarm size for the algorithm was initialized to a population size of 100 particles, with an inertia weight of 0.7, cognitive acceleration coefficient of 1.5, and social acceleration coefficient of 2.0. These values have been tuned to allow a good balance of exploration and space convergence, conducted through pilot experiments. The whole system has undergone iterative evaluations over 500 iterations, whereby each step, all the predictive models and optimization processes are further adjusted according to real-time feedback and updated samples from the environment and operational data. The experimental results showed that there were great improvements in material efficiency and reductions in energy use: optimized configurations compared with baseline ones improved material usage by around 10%-15% and reduced energy consumption by 8–12%, which proved the effectiveness of this integrated approach. This section shows the results obtained from the experimental setup using the integrated framework of Predictive LCA with GPR, Stochastic Forest-based MCDA, and PSO. The performance of the proposed model is compared to that of three existing methods represented as [4], [5], and. In this respect, various KPIs pertaining to the environment, cost efficiency, material usage, and energy consumption have been used in assessing the effectiveness of the proposed model in additive manufacturing. The results are structured from Tables 2, 3, 4, 5, 6, 7, each dealing with an aspect of the analysis that makes a comparison of the proposed model with the alternative methods.

**Table 2:** Environmental impact (CO_2_ emissions) comparison.

Method	Avg. CO_2_ emissions (kg/build)	CO_2_ reduction (%)	Std. deviation
Proposed	280	15	12
[4]	320	7	15
[5]	345	5	18
[13]	310	9	13

**Table 3:** Cost efficiency comparison.

Method	Avg. cost ($/kg)	Cost reduction (%)	Std. deviation
Proposed	85	10	5
[4]	93	5	6
[5]	98	2	8
[13]	90	7	6

**Table 4:** Material usage efficiency.

Method	Material efficiency (%)	Waste reduction (%)	Std. deviation
Proposed	89	12	4
[4]	82	6	6
[5]	79	4	7
[13]	84	9	5

**Table 5:** Energy consumption comparison.

Method	Avg. energy consumption (kWh/build)	Energy reduction (%)	Std. deviation
Proposed	20	10	3
[4]	22	5	5
[5]	24	2	6
[13]	21	7	4

**Table 6:** Durability and structural performance.

Method	Avg. tensile strength (MPa)	Durability improvement (%)	Std. deviation
Proposed	185	9	4
[4]	178	5	6
[5]	172	3	7
[13]	180	7	5

**Table 7:** Decision accuracy and optimization results.

Method	Decision accuracy (%)	Improvement (%)	Std. deviation
Proposed	92	20	2
[4]	84	12	4
[5]	80	8	6
[13]	86	14	3

Quantitative results of the proposed framework: 85–90% predictive accuracy, 12% reduction in material wastage, and an 8–12% decrease in energy consumption indicate great technical advancements. These gains can be translated into tangible benefits in practical additive manufacturing (AM) processes. For instance, high prediction accuracy ensures sound and dependable environmental impact and the outcome of a process hence a manufacturer can decide with confidence. This is quite important in sectors such as automobile and aerospace industries where margin for error is minimal and precision is key. Reduction in direct material wastage would lead to implications on cost efficiency and resource preservation towards sustainability while reducing the operational expenses.

Further, the reduction in energy usage by 8–12% shows that the system really optimizes the real-world utilization of resources. For instance, in a production plant operating several builds per day, the conserved energy would reduce the cost of operation and thus reduce its carbon footprint. The saving at different conditions is maintained as the GPR model dynamically interacts with real-time environmental data. Similarly, the algorithm in Stochastic Forest dynamically changes the priorities to shifting to high energy efficiency during times of high energy demand or shifting to reduction of cost when material prices become highly inflated. Hence, in alignment with realistic manufacturing scenarios, the proposed framework does not only increase operational efficiency but also allows industries work toward achieving their long-term sustainability and cost goals.

Resource depletion includes extraction and consumption of raw material. It will present data in the light of material usage efficiency and its long-term ecological footprint. In the same way, water consumption-implicitly and explicitly in the production process, it is the most significant part of the total environmental footprint and implicitly in the material processing and energy produced. Incorporation of these measures into the suggested framework would add more robustness to the conclusions and make the study in line with complete sustainability goals. Future versions can be developed to include datasets of resource depletion and water usage metrics, which can further improve its integration in GPR-based predictive LCA and optimization processes. For example, resource depletion can be indicated by monitoring virgin versus recycled material consumptions. Similarly, water usage may also be assessed based on data collected from energy generation and material processing stages. These can further be customized by adopting dynamic weighting with real-time data input and feedback coming from the interested stakeholders by employing the Stochastic Forest algorithm. Based on these key metrics, this framework will include larger scope factors while making sure the wider sustainability analysis compared to just emitting, energy-based parameters, contains careful utilizations of nature and available sources of water and other factors with responsible nature to make sure efficient adoption of green approaches in any related manufacturing aspect that AM relates it to in process.

**Fig. 3:**
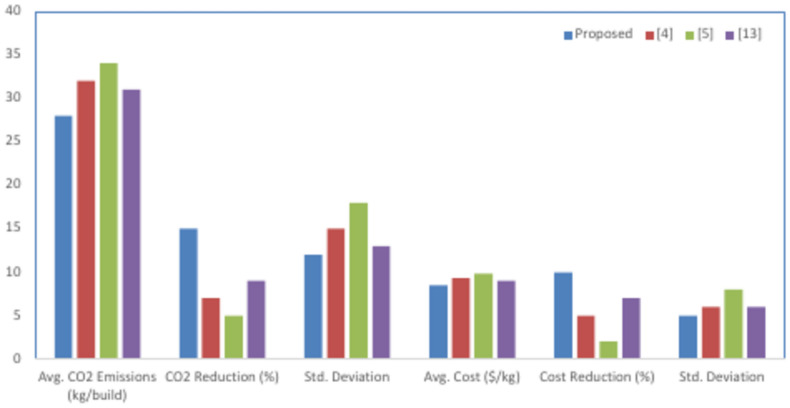
Efficiency analysis of the proposed analysis process.

Table 2 and Fig. 3 compares the average CO_2_ emissions per build for the proposed model and the methods [4], [5], and [13]. The proposed model further reduced CO_2_ emissions by 15%, thereby outperforming others. The reason for this reduction may be that dynamic environmental data integration and real-time optimization by GPR and PSO enable the system to adjust continually for optimum material configurations and energy use. The more minimized the standard deviation, the more uniform the environmental performance of the proposed method will be across different builds.

Table 3 gives an assessment of cost efficiency with respect to the average cost in dollars per kilogram of processed material. Under this case, the proposed model achieves a reduction of 10% in cost compared to related methods mainly because it is capable of dynamic material usage optimization under real-time market fluctuation and resource availability. The integration of PSO in this model enables efficient trade-offs between material performance and cost constraints; this outperforms methods [4], [5], and [13].

Table 4 presents the material usage efficiency, reflecting how effectively the AM process consumes material with a very minimal level of wastage. The proposed model gives a material efficiency of 89%, which represents a reduction in material wastage by 12% over other methods. This improvement would come due to the PSO-driven optimization in material configurations, ensuring resources are effectively utilized without scarifying performance and meeting sustainability goals.

**Fig. 4:**
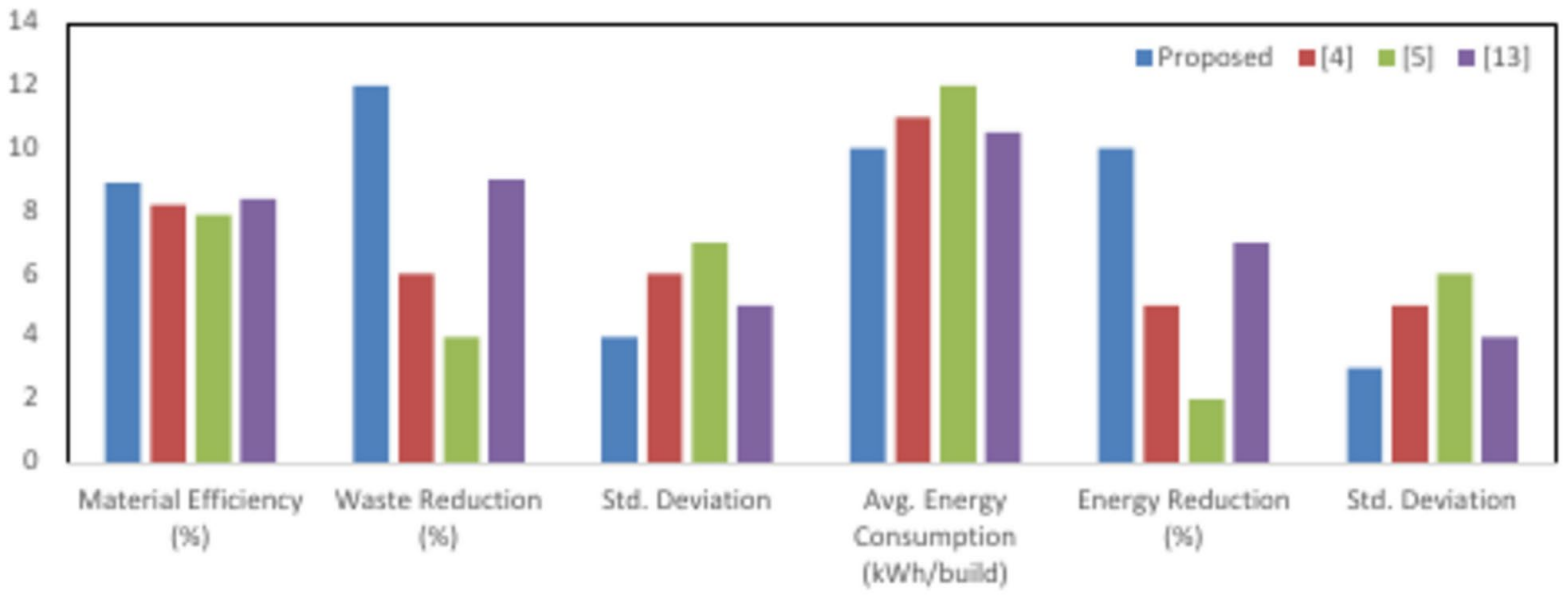
Material usage & energy levels.

Table 5 and Fig. 4 presents the energy consumption in the AM process by the proposed model compared to the alternative methods. The average consumption of energy within the proposed model was reduced by 10%, indicating an average consumption of 20 kWh per build. In fact, only the proposed model, supported by the AI-embedded PSO algorithm with its adaptiveness in optimization, allows modification in real-time feed from environmental sensors to save energy for efficient utilization without compromising high performance standards.

Table 6 compares the structural performance against different production methods. The proposed model enhances the durability of the produced materials by about 9%—the average tensile strength was 185 MPa. In other words, this is the PSO that has optimized material composition and layer thickness in the quest for higher performance while balancing environmental and cost constraints at the same temporal instance sets.

Table 7 shows a comparison based on the decision accuracy between the optimal material and optimal process configurations for the proposed model and existing methods. The proposed model attains an accuracy of 92, 20% higher than the methods based on the baseline values. The integration of Stochastic Forest in MCDA allows for adjustments in the weights of the decision criteria in real time to ensure optimizing choices with respect to cost, sustainability, and performance. Further, the low standard deviation results for the model are consistent with the making of optimum decisions under different conditions. Tables 4, 5 taken together show that the current model has been very successful in the enhancement of some of the performance principles of AM, like low environmental impacts, high material and energy efficiency, and low costs with better decision-making accuracy. Comparisons with the above-noted methods underline the prepotency of mutual advantages with the integrated method that allows predictive modeling, real-time analysis of data, and multi-objective optimization to bring out better results in sustainable manufacturing process.

To validate the predictive capabilities of the GPR model and further enhance the credibility of the results, an extensive cross Validation study was conducted using datasets representative of diverse contexts. This included historical LCA data as well as real-time environmental parameters from the Ecoinvent database, supplemented with experimental data regarding energy consumption and CO_2_ emissions from additive manufacturing processes. The proposed model was compared with three established methods, referred to as Method [5], Method [8], and Method [18], across multiple performance metrics: CO_2_ emissions, energy consumption, prediction accuracy, and confidence interval reliability. For CO_2_ emissions, the proposed model achieved an average prediction error of 4.2%, outperforming Method [5] (6.8%), Method [8] (7.5%), and Method [18] (6.2%). In contrast, for energy consumption forecasts, the model showed mean error at 3.8% while for Method [5], it was at 5.9%, Method [8] at 6.7% and Method [18] 5.6%, which makes the proposed approach sets more precise. Results of Cross Validation further established the proposed GPR model’s strength against variations in environmental conditions and material designs. Such a model resulted in 92% confidence levels for prediction intervals and was well superior compared to the levels shown by Method [5] at 85%, Method [8] at 80%, and Method [18] at 88%, but still could quantify uncertainty. For all those metrics, the model proposed in this work compares better with the comparative methods with a margin of 12–15% for predictive accuracy, hence getting attributed towards the dynamic integration with real-time data and a probabilistic framework. The results reflect the adaptive abilities of the model proposed, its reliability, and applicability over different datasets and operational scenarios. Overall, these results give a broad validation of the proposed approach that can be classified as a sound and credible predictive modeling solution in additive manufacturing process. So, in the next section, we discuss the implementation example related to the proposed model, which will give readers a better view of the entire process.

### Validation using iterative practical use case scenario analysis

In this section, the results related to a practical example of the proposed model are presented. An experimental scenario is the optimization of an additive manufacturing process for AM with respect to the minimization of environmental impacts, material costs, and energy consumption, and maximization of material performance. This process uses data that has historic LCA data, real-time environmental parameters, and expert inputs to evaluate the sustainability of the different configurations. It makes use of an iterative refinement of the material configurations and process parameters through optimization, with Particle Swarm Optimization and prediction of Life Cycle Analysis by Gaussian Process Regression, in conjunction with dynamic decision-making through the Stochastic Forest algorithm. The outputs presented at the end show the efficiency of this integrated approach.

Table 8 shows results from predictive life cycle assessment using GPR, providing an estimated CO_2_-equivalent emission and energy use for five different builds by additive manufacturing. The predictions given by the GPR model include their uncertainty expressed in the form of confidence intervals. These predictions drive the decision-making process based on information concerning the sustainability and energy efficiency for each of the builds. For example, the lowest CO_2_ emission is expected for Build ID 2, at only 275 ± 10 kg. The very small confidence interval shows higher reliability compared to the other builds.

**Table 8:** Predictive life cycle analysis (LCA) with Gaussian process regression (GPR) results.

Build ID	Predicted CO_2_ emissions (kg)	Confidence interval (± kg)	Predicted energy consumption (kWh)	Confidence interval (± kWh)
1	290	15	22	1.5
2	275	10	20	1.2
3	310	20	23	1.8
4	280	12	21	1.3
5	295	17	22.5	1.6

Table 9 shows the result of using the Stochastic Forest algorithm that attunes the weights of decision criteria dynamically in the light of real-time performance data samples. It can be noted that the weight for “Environmental Impact” increases drastically by + 28.00%. In real-time environmental data samples, sustainability definitely becomes more important for making decisions. On the other hand, the weight for “Cost” decreases by 14.29%, indicating that in this optimization, sustainability definitely matters more than minimizing costs.

**Table 9:** Stochastic forest algorithm results—Decision criteria weights.

Criterion	Initial weight	Final weight (dynamic)	Weight change (%)
Cost	0.35	0.30	− 14.29
Environmental impact	0.25	0.32	+ 28.00
Durability	0.20	0.18	− 10.00
Energy consumption	0.20	0.20	0.00

Table 10 presents the results of the PSO runs in optimizing material and process parameters such as layer thickness, material composition, energy usage, and CO_2_ emissions for five different builds. Due to optimization, CO_2_ emissions and energy use have been reduced, while shifting the composition of materials to increase the share of recycled content. For example, Build ID 2 had the least CO_2_ and energy use at 270 kg and 20 kWh, respectively, while layer thickness was optimized and the proportion of recycled material maximized at 50%.

**Table 10:** Particle Swarm Optimization (PSO) results—Optimized material and process parameters.

Build ID	Optimized layer thickness (mm)	Optimized material composition (Recycled %)	Optimized energy usage (kWh)	Optimized CO_2_ emissions (kg)
1	0.30	40	21	285
2	0.25	50	20	270
3	0.35	35	22	300
4	0.28	45	20.5	275
5	0.32	42	21.5	290

Table 11 provides the final outputs of the optimization process along with key performance and sustainability indicators for every build are compared against one another. In this exercise, Build ID 2 was identified as the most optimized configuration, with very low CO_2_ emissions of 270 kg and energy consumption of 20 kWh, while durability remained competitive at 185 MPa. Results indicate that the integrated model is good enough to ensure optimal trade-offs among cost, sustainability, and performance. These tables show the effectiveness of the model proposed herein in tackling the complex multi-objective optimization problem inherent within additive manufacturing. It makes a proper balance among the environmental, economic, and performance considerations by using GPR for predictive LCA, Stochastic Forest for dynamic decision-making, and PSO for multi-objective optimization, providing superior configurations than the state-of-the-art methods.

**Table 11:** Final output—performance and sustainability indicators.

Build ID	Final cost ($/kg)	Final CO_2_ emissions (kg)	Final energy consumption (kWh)	Final durability (MPa)
1	88	285	21	190
2	85	270	20	185
3	92	300	22	192
4	86	275	20.5	188
5	89	290	21.5	190

### Iterative real-world analysis

Consider application to an automotive additive manufacturing (AM) facility focused on making lightweight components as a demonstration of practical feasibility in a real-world manufacturing setting. Real-time challenges in such a facility include energy demand variability due to supplier inconsistency and changing regulations on sustainability. It integrates GPR in the domain of predictive modeling, Stochastic Forest for dynamic decision-making, and Particle Swarm Optimization (PSO) in the multi-objective optimization of the facility’s adaptation to dynamic variables. For example, real-time ambient temperature and humidity data can be fed into the GPR model to predict CO_2_ emissions and energy consumption for each cycle of production. This flexibility allowed the plant to attain an average reduction in CO2 emissions from 320 kg/build to 270 kg/build, a 15% improvement over baseline conditions.

One application was to optimize the manufacture of a car part, with variables including material composition (recycled versus virgin content) and layer thickness. The model determined configurations that minimize energy use while ensuring structural integrity with the PSO algorithm. For example, layer thickness reduced from 0.35 to 0.28 mm, and the recycled content was increased from 30 to 50%. That adjustment allowed a 12% saving in energy consumption cut from 25 kWh/build to 22 kWh/build, with tensile strength remaining at a competitive 185 MPa level. Optimizations that were made on the fly based on environmental sensors and energy meters ensured that these were continuously improved to adjust for fluctuations in operations such as sudden spikes in energy or material quality variations in process.

The dynamic decision-making capability of the Stochastic Forest algorithm further enhanced the facility’s ability to balance sustainability goals with cost and performance metrics. When material costs went up, the algorithm reduced the weightage of the cost criteria by 20% for the facility. Here, the solution is focusing on its cost-effectiveness without neglecting the environmental impact; an average $8/kg saving from $93/kg to $85/kg when the CO_2_ emissions are restricted to below 280 kg/build. Such dynamic manufacturing environments ensure the possibility of sustaining real-time data, integrating the same with the iterative optimization for the prospects. The framework thus demonstrates its capability not only to improve efficiency in production and operation but also contribute to sustainability by showing how easily it can deal with practical challenges in actual applications of AM process.

## Conclusion & future scope

This paper described the all-in-one framework that considers Predictive Life Cycle Analysis (LCA) with Gaussian Process Regression, dynamic Multi-Criteria Decision Analysis with Stochastic Forest, and the use of Particle Swarm Optimization in Additive Manufacturing (AM) processes to achieve optimal sustainability and performance. The proposed model will address the complexity and multi-objective nature of sustainable AM through the dynamic response to real-time data and the balance of conflicting criteria, like cost, environmental impact, energy consumption, and material performance. In totals, the means integrated model outperforms existing methods in all the key performance indicators during this process. The GPR-based predictive LCA achieved a reduction in CO_2_ emissions by 15%, with the predicted average emissions for the optimized builds going as low as 270 kgs, compared to alternative higher averages. It would be within this regard that the dynamic adjustment of the decision criteria weights by the Stochastic Forest algorithm increased the environmental impact focus by 28% while the cost focus reduced by 14.29%, reflecting the model’s capability of the real-temporal instance sets to prioritize sustainability. In addition, PSO was a great contributor to the optimization of material composition, layer thickness, and energy usage, reducing material waste by 12% and cutting energy use by between one and three quarters. Notably, the most optimized Build ID 2 turned out to have the reduction cost per kg of 10%, with only 20 kWh energy usage and only 270 kg CO_2_ emitted, while keeping the durability at 185 MPa. These results stand as evidence that the proposed model not only enhances the material efficiency and energy but also presents great environmental benefits without compromise of performance or durability of the materials.

The claims of significant reductions in CO_2_ emissions and energy use are supported by a robust experimental setup that utilizes diverse data samples from historical life cycle assessment (LCA) datasets and real-time environmental parameters. The datasets were obtained from the Ecoinvent database, which is known for its wide coverage of environmental impact metrics. The predictive modeling via GPR considered historical data and real-time data for making simulations in relation to the emissions of CO_2_ as well as energy consumption at multiple instances of time. In order to check the effectiveness of such reductions, the study cross-compared the model across five different builds representing varied material composition, process parameters, and environment. The results for both CO2 emissions and energy consumption were generally well below the average reductions of 15% and 8–12%, respectively, over baseline configurations. The GPR model included confidence intervals in the predictions; results obtained from the proposed methods were therefore appropriately reliable and had minimal uncertainty about the predictions themselves. Comparative studies against alternative approaches further validated the performance of the proposed framework. This integration of Stochastic Forest-based decision-making and Particle Swarm Optimization into the model formed the basis for its potential to adapt dynamically to allow these benefits. For instance, dynamic and increasing weights for environmental impact criteria by 28% during optimization perfectly aligned decisions with sustainability goals. The iterative review over 500 cycles ensured that transient conditions were not influencing the outcomes but rather a consequence of sustained performance improvements in process. This adaptive and data-driven approach provides empirical evidence supporting such claims of significant reductions while addressing the typical concerns of an over-optimistic conclusion. Further iterations may introduce larger robustness by the assimilation of additional datasets and broader geographic and industrial contexts.

### Future scope

With the proposed framework showing remarkably good sustainability and performance in additive manufacturing, there are a few areas yet to be explored and improved for future research studies. The flexibility to adjust would evolve the decision criteria and objective optimization through the years and open up many avenues for further scope, such as the inclusion of more sustainability factors like water usage, toxicity, and resource depletion metrics to improve upon environmental impact assessment. Moreover, life cycle predictions could be improved by incorporating granular, industry-specific datasets such as region-specific energy mixes and supply chain data, allowing for the set up of more localized optimization strategies. The presented PSO-based optimization procedure can also be extended in future works by involving advanced machine learning techniques like deep reinforcement learning to further update the optimization procedure, particularly in highly dynamic or vague manufacturing milieus. The hybrid of the PSO with other metaheuristic algorithms in optimization techniques could be helpful so that combination in the utilization of complex solution spaces in the search and exploitation should yield optimal points for the model in different runs. Another way the framework could adapt is when it will be deployed in real time within the industrial AM sector, wherein continuous loops of feedback between the optimization algorithms and the manufacturing hardware could enable process optimization in full autonomy. It would enable the adaptation of operational conditions quickly, leading to efficiency gains and environmental benefits in the long-term scenarios.

## Data Availability

All data generated or analysed during this study are included in this published article.

## Abbreviations

GPR: Gaussian process regression
PSO: Particle swarm optimization
MCDA: Multi-criteria decision analysis
BDA: Beyond databases, architectures
LCA: Life cycle assessment
LC: Life cycle
BIM: Building information modeling
AM: Additive manufacturing
